# *Mycobacterium chimaera *pulmonary infection complicating cystic fibrosis: a case report

**DOI:** 10.1186/1752-1947-5-473

**Published:** 2011-09-22

**Authors:** Stéphan Cohen-Bacrie, Marion David, Nathalie Stremler, Jean-Christophe Dubus, Jean-Marc Rolain, Michel Drancourt

**Affiliations:** 1 Unité de Recherche sur les Maladies Infectieuses et Tropicales Emergentes, CNRS-UMR 6236, IRD 198, IFR 48, Faculté de Médecine, Université de la Méditerranée, 27, Boulevard Jean Moulin, Marseille cedex 5, France; 2 Assistance Publique des Hôpitaux de Marseille-Pôle des Maladies Infectieuses, Hôpital la Timone, rue Saint-Pierre 13005 Marseille, France; 3 Département des Maladies Respiratoires, Centre de Ressources et de Compétences pour la Mucoviscidose (CRCM), Hôpital Timone Enfants, rue Saint-Pierre 13005 Marseille, France

## Abstract

**Background:**

*Mycobacterium chimaera *is a recently described species within the *Mycobacterium avium *complex. Its pathogenicity in respiratory tract infection remains disputed. It has never been isolated during cystic fibrosis respiratory tract infection.

**Case presentation:**

An 11-year-old boy of Asian ethnicity who was born on Réunion Island presented to our hospital with cystic fibrosis after a decline in his respiratory function over the course of seven years. We found that the decline in his respiratory function was correlated with the persistent presence of a *Mycobacterium avium *complex organism further identified as *M. chimaera*.

**Conclusion:**

Using sequencing-based methods of identification, we observed that *M. chimaera *organisms contributed equally to respiratory tract infections in patients with cystic fibrosis when compared with *M. avium *subsp. *hominissuis *isolates. We believe that *M. chimaera *should be regarded as an emerging opportunistic respiratory pathogen in patients with cystic fibrosis, including young children, and that its detection warrants long-lasting appropriate anti-mycobacterial treatment to eradicate it.

## Introduction

*Mycobacterium avium *complex (MAC) organisms are opportunistic pathogens, and their isolation from the respiratory tract of cystic fibrosis (CF) patients correlates with declining respiratory function [1]. Six of nine MAC species have been described in the past five years [2-4], but the spectrum of MAC organisms infecting CF patients is not well established; only *M. avium *and *Mycobacterium intracellulare *have been reported in these patients [1,5].

Our reference mycobacteriology laboratory routinely processes respiratory tract specimens collected from all CF patients who are followed by the Cystic Fibrosis Regional Reference Center in Marseilles, France. After Ziehl-Neelsen staining, cultures were routinely performed in BACTEC broth using the BACTEC 9000 MB system (BD Biosciences, Sparks, MD, USA), subcultures were grown on 5% sheep blood agar, and identification was done by partial *rpoB*-based sequencing [2]. Genotyping of the isolates was performed using multi-spacer sequence typing (MST) [6]. The minimum inhibitory concentrations were determined by 10-day incubation of a 1 McFarland mycobacterial suspension at 37°C on 5% sheep blood agar in the presence of the respective Etest strip (AB Biodisk, Solna, Sweden). The susceptibility to rifabutin was determined in BACTEC broth using the BACTEC 9000 MB system by incubating a 1 McFarland bacterial suspension with 1 μg/mL or 2 μg/mL rifabutin for five days in the presence of a growth control.

Over a six-year period, five (4.3%) of 115 children with CF had at least one respiratory tract specimen that was positive for MAC. *M. avium *subsp. *hominissuis *was found in two patients (with distinct MST48 and MST20 genotypes), *Mycobacterium colombiense *was found in one patient, and *Mycobacterium chimaera *was found in two additional patients (distinct MST33 and MST39 genotypes).

### Case presentation

**Figure 1:**
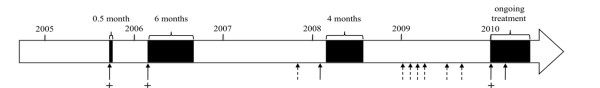
**Chronological representation of *Mycobacterium chimaera *respiratory tract infection in a CF patient**. Arrows indicate requests for mycobacterial detection in the sputum. Dotted arrows indicate negative culture. Filled arrows indicate positive culture for *M. chimaera*. + indicates acid-fast bacilli stain seen upon direct microscopic examination of sputum samples. Antibiotic therapy is represented by black squares. Duration of treatment (in weeks) is indicated above the brackets.

One of the latter five patients with CF described at the end of the Introduction was an Asian boy born on Réunion Island who exhibited a ΔF508 homozygous deletion in the *CFTR *gene, which is diagnostic of CF. He presented to our hospital when he was 11 years old because he had started complaining of hemoptoic cough. A physical examination revealed weight loss and wheezing. After his arrival in metropolitan France, a MAC organism further identified as genotype ST33 *M. chimaera *was isolated from his respiratory tract (Figure 1). This isolate was susceptible to clarithromycin (3 μg/mL), rifabutin (< 1 μg/mL), and ethambutol (2 μg/mL) and was resistant to ofloxacin (> 32 μg/mL), streptomycin (> 1024 μg/mL), doxycyclin (> 256 μg/mL), and rifampicin (> 32 μg/mL). The patient was given rifabutin and clarithromycin, but he interrupted this treatment after two weeks. Six months later his sputum yielded acid-fast bacilli that were identified as genotype MST33 *M. chimaera *after culture. The same antibiotic regimen prescribed for six months yielded negative mycobacterial cultures. Two years later a recurrent fever and cough correlated with MST33 *M. chimaera-*positive cultures. The patient once again interrupted his therapy of rifabutin combined with clarithromycin, this time after four months. Twenty-two months later the patient's condition severely worsened, as he had developed chronic respiratory insufficiency exacerbated by bilateral pneumothorax, which required non-invasive ventilatory support. From this period onward, insulin therapy became necessary to achieve glycemic control, and his nutritional condition justified the initiation of enteral feeding by percutaneous gastrostomy. The thoracic computed tomographic scan showed bronchiectasis, expanded areas of condensation, and mediastinal polyadenopathy. Acid-fast bacilli that were observed on sputum smears were cultured and revealed to be MST33 *M. chimaera *organisms. Rifabutin and clarithromycin were reintroduced, but sequential cultures remained positive. The patient was registered in the national list of lung transplant candidates.

During the whole treatment period, the microbiological investigations of the respiratory tract specimens also exhibited *Pseudomonas aeruginosa*, methicillin-susceptible *Staphylococcus aureus*, *Stenotrophomonas maltophilia*, and *Aspergillus fumigatus*.

## Discussion

The prevalence of MAC organisms in patients with CF is highly variable, ranging from 9% in the US [1] to 1.5% in France, and it was evaluated at 1.8% in southeast France [5], where Marseilles is located, although our data yielded a prevalence of 4.3% there. In France and the US, *M. avium *has contributed to twice as many positive samples as *M. intracellulare *[5,7]. However, the identification of MAC organisms has relied on hybridization probe-based methods, which has misidentified *M. chimaera *as *M. intracellulare *[3], thus preventing the detection of *M. chimaera *in patients with CF. Using sequence-based identification, we observed that *M. chimaera *organisms contribute equally to respiratory tract infections in patients with CF compared with *M. avium *subsp. *hominissuis *isolates.

The pathogenicity of *M. chimaera *remains controversial. It has been reported to cause respiratory infections in patients with pulmonary diseases other than CF, including chronic obstructive pulmonary disease, bronchiectasis, and cavitation [3]. Conversely, in two studies with an aggregate of 97 non-CF patients OK, only 3.3% infected with *M. chimaera *fulfilled the American Thoracic Society (ATS) criteria for MAC lung disease [8,9]. The patient described in our case report eventually fulfilled the ATS criteria on the basis of positive cultures from at least two separate sputum samples. Moreover, his *M. chimaera *infection relapsed every time antibiotic treatment was interrupted, and this continuing infection correlated with a worsening condition of the patient in the presence of associated pathogens, which may have also contributed to this downward spiraling evolution [10].

## Conclusion

We believe that *M. chimaera* should be regarded as an emerging opportunistic respiratory pathogen in patients with CF, including young children, and that its detection warrants long-lasting, appropriate antimycobacterial treatment to eradicate it.

## Abbreviations

ATS: American Thoracic Society; CF: cystic fibrosis; MAC: *Mycobacterium avium *complex; MST: multi-spacer sequence typing.

## Consent

Written informed consent was obtained from the patient's mother for publication of this case report and any accompanying images. A copy of the written consent is available for review by the Editor-in-Chief of this journal.

## Competing interests

The authors declare that they have no competing interests.

## Authors' contributions

SCB, JMR, JCD, and MD reviewed the clinical data and were major contributors to the writing of the manuscript. MD and NS were involved with patient management. SCB, JMR, and MD analyzed data and performed the laboratory analysis. All authors read and approved the final manuscript.
